# Enhanced Detection of Estrogen-like Compounds by Genetically Engineered Yeast Sensor Strains

**DOI:** 10.3390/bios14040193

**Published:** 2024-04-15

**Authors:** Nidaa Abu-Rmailah, Liat Moscovici, Carolin Riegraf, Hadas Atias, Sebastian Buchinger, Georg Reifferscheid, Shimshon Belkin

**Affiliations:** 1 Institute of Life Sciences, The Hebrew University of Jerusalem, Jerusalem 91904, Israel; nidaa.aburmalah@mail.huji.ac.il (N.A.-R.); liatmosc@savion.huji.ac.il (L.M.); hadas.atias@mail.huji.ac.il (H.A.); 2 Federal Institute of Hydrology (BfG), Department Biochemistry, Ecotoxicology, 56068 Koblenz, Germany; riegraf@bafg.de (C.R.); buchinger@bafg.de (S.B.); reifferscheid@bafg.de (G.R.)

**Keywords:** high-performance thin-layer chromatography (HPTLC), endocrine-disrupting compounds (EDCs), enhanced green fluorescent protein (EGFP), wastewater treatment plants (WWTPs), yeast-based estrogen bioreporters

## Abstract

The release of endocrine-disrupting compounds (EDCs) to the environment poses a health hazard to both humans and wildlife. EDCs can activate or inhibit endogenous endocrine functions by binding hormone receptors, leading to potentially adverse effects. Conventional analytical methods can detect EDCs at a high sensitivity and precision, but are blind to the biological activity of the detected compounds. To overcome this limitation, yeast-based bioassays have previously been developed as a pre-screening method, providing an effect-based overview of hormonal-disruptive activity within the sample prior to the application of analytical methods. These yeast biosensors express human endocrine-specific receptors, co-transfected with the relevant response element fused to the specific fluorescent protein reporter gene. We describe several molecular manipulations of the sensor/reporter circuit in a *Saccharomyces cerevisiae* bioreporter strain that have yielded an enhanced detection of estrogenic-like compounds. Improved responses were displayed both in liquid culture (96-well plate format) as well as in conjunction with sample separation using high-performance thin-layer chromatography (HPTLC). The latter approach allows for an assessment of the biological effect of individual sample components without the need for their chemical identification at the screening stage.

## 1. Introduction

A wide range of chemicals are released into the aquatic environment through diverse sources, including wastewater treatment plants, landfills or agricultural activities. Some of these chemicals possess endocrine-like properties which may mimic, disturb or block the endogenous endocrine system in humans and wildlife. The harmful effects of these natural or synthetic chemicals, collectively grouped as endocrine-disrupting compounds (EDCs), have been demonstrated at the ng/L and even pg/L range [1,2].

Wastewater is often contaminated with EDCs originating from hospital, industrial, agricultural and domestic wastes. Removal of EDCs from raw sewage in wastewater treatment plants (WWTPs) by physical, chemical and biological processes is, in many cases, incomplete. Consequently, such compounds have been reported to be regularly detected in both surface and groundwater [1,3,4,5] as a result of the discharge of wastewater effluents into the environment or their use for irrigation.

Common analytical methodologies for EDC detection and identification, such as high-performance liquid chromatography (HPLC) or mass spectrometry (MS), are highly sensitive and accurate [1,3]. These analytical methods, however, are restricted to a relatively limited list of known chemicals previously demonstrated to affect the endocrine system. This hinders the detection and identification of other chemicals present in the environment, including those with potential endocrine-disrupting activities. In addition, many new chemicals, introduced in response to changing industrial needs and consumer demands, continuously find their way to the environment [5]. This regulatory weakness has created an acute need for the effective monitoring of EDCs in natural samples, with no a priori requirement for their chemical identification.

This need may be at least partially fulfilled by effect-based methods, which provide a complementary approach to analytical techniques by detecting the biological activity of a sample or of its individual components, rather than analyzing its exact composition [1,6,7,8,9,10,11]. In most cases, these methods are based on the use of whole-cell bioreporters, genetically engineered to detect EDCs by providing a selective and dose-dependent quantitative signal in their presence. Such live bioreporters of bioactive analytes can be employed in a preliminary screening assay, identifying samples that need to be further subjected to chemical analysis [12]. In response to the high diversity of suspected EDCs in the environment, we have previously demonstrated the multi-parallel detection of estrogenic and androgenic compounds using *Saccharomyces cerevisiae*-based bioreporters expressing different fluorescent proteins in response to different EDC classes [7]. These fluorescent sensor cells harbored a human endocrine receptor gene (hER, estrogen receptor, or hAR, androgen receptor), under the control of a constitutive strong promoter, integrated into the yeast genome. A plasmid-borne fluorescent reporter gene was fused downstream of repetitive estrogenic or androgenic hormone response element (HRE) sequences, creating a hairpin structure which repressed reporter gene expression. When the ligand–receptor complex binds to the HRE sequences, the hairpin structure is released and the fluorescent reporter gene is expressed [13]. That study provided a proof-of-principle for the parallel detection of androgenic and estrogenic effects using a co-culture of fluorescent bioreporters [7]. Beatz et al. [14] have similarly demonstrated multi-parallel EDC detection by the use of fluorescent *Arxula adeninivorans* yeast sensor strains; in both cases, the bioassay was successfully combined with sample separation by HPTLC.

In the present study, we have investigated several molecular options to enhance the performance of fluorescent EDC sensor strains. Using the estrogen-sensing construct as a model bioreporter, we have introduced mutations into the host cell and have molecularly manipulated both the reporting element and the promoter that drives its expression. Host strain modifications included the deletion of three ATP-binding cassette (ABC) multidrug transporters, a large superfamily of membrane protein complexes that are able to couple the energy yielded by ATP hydrolysis to the active transport of substrates across the membrane [15]. The three deleted genes from the host *S. cerevisiae* genome, comprising the pleiotropic drug resistance network [16], were PDR5 (involved in resistance to xenobiotic compounds and cations and in steroid transport), SNQ2 (in addition to multidrug resistance, involved in resistance to singlet oxygen species; its disruption confers sensitivity to 4-nitroquinoline-N-oxide) and YOR1 (which exports oligomycin, organic anions and diverse additional compounds). Deletion of these genes has previously been reported to generate sensitive strains suitable for high-throughput drug screening [17]. In addition, to tighten and better control reporter gene expression, we have modified the original plasmid by replacing the strong constitutive GPD and ADH1 yeast promoters with a minimal CYC1 promoter [7]. We have also introduced an EGFP reporter gene in either a single or a double copy, and have deleted a superfluous *luxA* gene.

## 2. Materials and Methods

### 2.1. Chemicals

Both 17β-estradiol (E2, CAS: 50-28-2) and 17α-ethinylestradiol (EE2, CAS: 57-63-6), used as model estrogenic reference compounds, were of the highest analytical grade and were purchased from Sigma-Aldrich. Stock solutions of the reference compounds (5 mg/mL) were prepared in ethanol. Chromatographic separation was performed on silica gel HPTLC plates of type 60G F254 (20 × 10 cm or 10 × 10 cm) purchased from Merck (Darmstadt, Germany). HPTLC solvents were of the highest analytical grade and were purchased from Merck.

### 2.2. Yeast Strains, Plasmids and Growth Conditions

Strains and plasmids used in this study are listed in Table 1. *Escherichia coli* DH5α (NEB, HIT competent cells), used as a host for plasmid construction and maintenance, was grown in lysogeny broth (LB) at 37 °C with or without ampicillin (100 µg/mL), depending on the requirement for plasmid maintenance.

**Figure 1:**
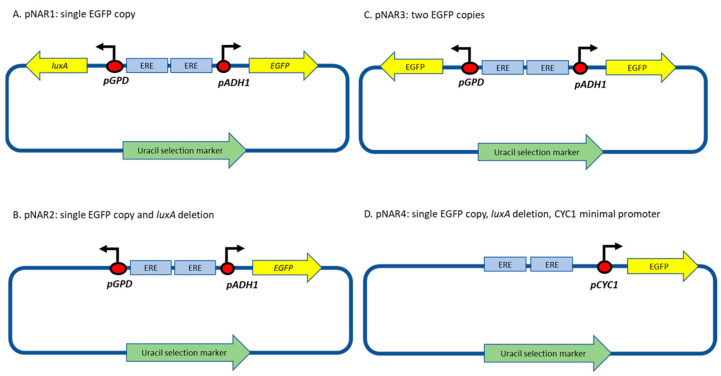
Schematic design of the estrogen-responsive fluorescent plasmids constructed in the course of this study. All versions contain two copies of the human estrogen response element (EREs). Upon binding of a receptor–ligand complex to the response elements, a hairpin structure is released and activation of the adjacent promoters is enabled, resulting in the transcription of the downstream genes.

A previously constructed *S. cerevisiae* sensor strain and plasmid [7] was employed in this study as a basis for the construction of the new fluorescent bioreporters. Yeast strain hER, harboring the human estrogen nuclear receptor integrated into the yeast genome, was purchased from BioTech (Knoxville, TN, USA). Plasmid pUTK407 [13] was kindly donated by Prof. S. Ripp (University of Tennessee, Knoxville, TN, USA). This plasmid contained the *Photorhabdus luminescens luxA* and *luxB* genes, downstream to the strong constitutive promoters GPD and ADH1, respectively. The plasmid also harbors two repetitive sequences of the human estrogen hormone response element (HRE) fused upstream to the two divergent promotors, forming a hairpin structure that represses activation of both promoters. In the original plasmid, upon binding of the ligand–receptor complex to its respective HRE, this hairpin structure was released, and both *luxA* and *luxB* were divergently transcribed, yielding the two structural subunits of the bacterial luciferase. In a previous study [7], the *luxB* gene was replaced by a yeast-optimized sequence coding for the enhanced green fluorescent protein (EGFP), yielding plasmid pNAR1 (Figure 1A), which served as the basis for the current study.

In the present study, plasmid pNAR1 has been modified in the following manners (PCR primers are listed in Supplementary Table S1):

1. Deleting the *luxA* gene, yielding plasmid pNAR2 (Figure 1B). Plasmid NAR1 was digested with *NotI* and *SalI* restriction enzymes, and a Klenow fragment (New England Biolabs (Ipswich, MA, USA)) was used to create blunt ends, allowing the ligation of the complementary sequence.
2. Substituting both the *luxA* and *luxB* genes with identical EGFP genes, to produce plasmid pNAR3 (Figure 1C), using restriction enzymes followed by ligation to a respective insert fragment.
3. Replacing the ADH1 promoter with the minimal cytochrome C promoter (CYCmin; Plasmid pNAR4, Figure 1D). CYCmin was amplified from plasmid Prsii426-ERE-yNLucP [6], a kind gift from Prof. E. Michelini and Dr. A. Lopreside (University of Bologna, Bologna, Italy), and inserted instead of the ADH1 promoter using the Gibson assembly technique (NEBuilder HiFi DNA Assembly Cloning kit, New England Bio Lab) [18], employing the primers listed in Table S1.

The *S. cerevisiae* strains harboring plasmids pNAR1 to pNAR4 were designated NAR1 to NAR4, respectively.

### 2.3. Deletion of Three Plasma Membrane ABC Cassette Transporter Genes

The permeability of the host strain’s membrane was modified by the deletion of three plasma membrane transporter genes from the genome of the hER strain, PDR5, SNQ2 and YOR1 [16,19], employing the cre-lox procedure [20]. The target genes were replaced with the kanMX module, conferring kanamycin resistance, which was removed from the genome using the loxP-kanMX-loxP gene disruption cassette from plasmid pUG36 [20], a gift from Prof. M. Schuldiner (Weizmann Institute of Science, Rehovot, Israel). Briefly, linear DNA was generated by PCR amplification of the loxP-KAN-loxP cassette using primers 1–6 (Table S1), each primer containing a sequence homologous to one of the three transporters. The hER strain was transformed first with the *pdr5*:KAN disruption cassette and plated on yeast extract–peptone–dextrose (YPD) plates containing 200 µg/mL of the aminoglycoside antibiotic geneticin (G418). Genomic DNA was extracted from hER *pdr5*:KAN colonies using the DNA-Pure yeast genomic kit (CPG Inc., Lincoln Park, NJ, USA), confirmed by PCR and sequenced. Kanamycin-resistant mutants were transformed with plasmid pBF3038 [20], carrying the Cre-recombinase gene under the control of the galactose-inducible GAL1 promoter with a leucine selective gene, and plated on yeast complete medium plates lacking leucine. Expression of the Cre-recombinase enzymes was induced by shifting cells from a leucine-free medium with 2% glucose to the same medium with 2% galactose. The cells were re-grown for 4 h under the same conditions and then plated on YPD plates. Following overnight growth, the plates were replica-plated onto YPD containing geneticin (200 µg/mL). Colonies that lost the kanMX gene (i.e., did not grow on geneticin plates) were isolated from the YPD plates; gene deletion, leaving behind a single loxP site, was verified by colony PCR and sequencing. Using the appropriate primers (Table S1), this process was repeated for the other two transporters, generating a host strain with the three desired knockouts. The triple-mutant sensor strain harboring pNAR4 was designated NAR5.

### 2.4. Estrogenicity Assay, 96-Well Plates

Yeast strains were grown overnight at 30 °C with agitation (200 rpm) in a selective medium lacking uracil (unless noted otherwise). The culture was then diluted 100-fold in the same medium and re-grown under the same conditions to the late exponential growth phase (OD_600_ = 0.6–1), following which the cells were exposed to the tested sample in an aqueous solution in a 96-well plate. The tests were performed with at least three independent cultures on different days using individual cryogenically preserved cultures. Aliquots (40 µL) from the diluted culture were added to all wells of a 96-well clear-bottom black microtiter plate (Greiner), containing 80 µL of model compounds, dissolved in 1% ethanol at predetermined concentrations. Ethanol (1%) was used as the negative control. The plates were incubated at 30 °C for 18 h and the fluorescent signal was measured every 2 h, following a 10 s vigorous shaking of the plate, in an Infinite M200 Pro plate reader (Tecan, Männedorf, Switzerland) using excitation/emission wavelengths of 488/507 nm. Fluorescence values are displayed as the instrument’s arbitrary relative fluorescence units (RFUs). Another parameter representing the response intensity was the response ratio, calculated as the fluorescence intensity in the presence of a known inducer or unknown sample divided by that of the uninduced control.

### 2.5. Estrogenicity Assay, HPTLC Plate

Thin-layer chromatography was performed on glass HPTLC plates, type 60G F254 (10 × 10 cm or 20 × 10 cm, Merck), as described before [7,21]. Briefly, the HPTLC plates were developed with methanol to 5 mm below the rim, dried at 120 °C for 30 min and stored in a desiccator at room temperature until used. Separation was performed in a glass development chamber by submerging the bottom of the HPTLC plate in a chloroform/acetone/petroleum (55:20:25) mixture up to 10 mm below the rim. The plates were then dried in a chemical hood until the organic solvents evaporated. For the detection of estrogenic activity on the HPTLC plate, 2 mL of an overnight fresh culture was sprayed homogenously on the developed HPTLC plate using a CAMAG (Muttenz, Switzerland) automated sprayer (CAMAG Derivatizer, spraying level 3, yellow nozzle). Following incubation of 4 to 18 h at 30 °C in an opaque plastic box, in which humidity was maintained by a water-soaked paper towel, the fluorescent EGFP signals were detected using a Fusion FX imaging system (Vilber, Marne-la-Vallée, France) at excitation/emission wavelengths of 365 nm/565 nm. For a quantitative evaluation of the fluorescence signals, the chromatogram was also documented using a TLC Scanner 4 (CAMAG).

### 2.6. Wastewater Treatment Plant Sample Preparation

Influent and effluent samples of municipal wastewater treatment plants (WWTPs) were collected and treated as described before [7,21,22]. Briefly, influent and effluent WWTP samples, collected and stored at 4 °C on the previous day, were centrifuged (Thermo Scientific, Sorvall RC6 Plus centrifuge, 17,000 RCF, 20 min) and the supernatant was filtered through a glass fiber filter (Pall, type A/C, Ø 47 µM). The filtered influent and effluent samples were enriched 200-fold and 500-fold, respectively, by solid-phase extraction (SPE) using Oasis HLB cartridges (200 mg, 6 mL; Waters Corporation, Milford, MA, USA). The extracts were stored in 1.5 mL amber vials at −20 °C until use. Different extract volumes (5–20 µL) were loaded onto the HPTLC plate, as in Section 2.4 above, according to the expected strength of the estrogenic effect.

### 2.7. Calculations

The limit of detection (LOD) was determined as the compound concentration equal to the sum of the average signal intensity of the noise (fluorescence in the inducer-free control) and three standard deviations of this noise. This was calculated for each strain by the following equation:

(1) $$\mathrm{LOD}\;(\mathrm{ng}/L)\;=\;{\overset{=}{x}}_{\mathrm{noise}}\;+\;3\times {std}_{noise},$$

where ${\overset{=}{x}}_{\mathrm{noise}}$ and *std_noise_* are the mean and the standard deviation of the noise, respectively.

## 3. Results

### 3.1. EGFP Synthesis in Response to Model Estrogenic Compounds in a 96-Well Plate Assay

**Figure 2:**
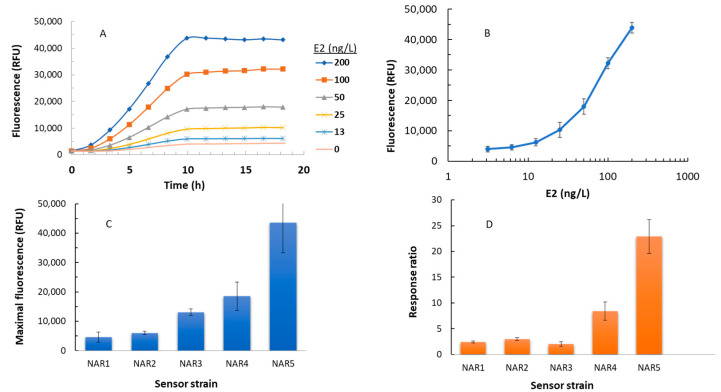
E2 detection performance of the five sensor strains. (**A**) Time-dependent fluorescent response of strain NAR5 to different E2 concentrations (representative experiment); (**B**) Maximal response to E2 of strain NAR5 over an 18 h exposure; (**C**) Maximal response intensity to E2 (200 ng/L) over an 18 h exposure of all 5 sensor strains; (**D**) Maximal response ratio to E2 (200 ng/L) over an 18 h exposure of all 5 sensor strains. All experiments were repeated at least three times, in duplicate.

In an attempt to increase the detection sensitivity of the yeast-based fluorescent EDC sensors, we molecularly manipulated both the host genome and the design of the plasmid-based reporter circuit, generating four new sensor variants. These were exposed to 17β-estradiol (E2), along with the control strain NAR1, at concentrations ranging from 3.125 ng/L to 200 ng/L. An example of the time-dependent response of strain NAR5 to different E2 concentrations is presented in Figure 2A, and the fluorescence intensity of that strain as a function of the E2 concentration is shown in Figure 2B. Figure 2C,D display the maximal fluorescence measured in the presence of a single E2 concentration (200 ng/L) and the response ratio to that concentration, respectively, of all five sensor strains. Table 2 summarizes the results presented in Figure 2, and presents the calculated limit of detection for all five variants.

The NAR2 strain, harboring a single copy of the EGFP gene and a deletion of the *luxA* gene, displayed an enhancement in signal intensity (ca. 30%) and response ratio (ca. 36%) compared to the parental NAR1 strain, but the limit of detection was not significantly affected. The presence of an additional EGFP in sensor strain NAR3 generated over a two-fold increase in the maximal signal intensity, but the response ratio and LOD values were actually inferior to both NAR1 and NAR2. Replacing the ADH1 promoter with the CYCmin promoter elicited a marked improvement in all three parameters, whereas the introduction of the three mutations (Δ*pdr5*, Δ*snq2*, and Δ*yor1*) yielded the lowest LOD value (8 ± 1 ng/L). The maximal fluorescent signal intensity and response ratio were also significantly enhanced in NAR5, both by over nine- fold

### 3.2. Estrogenicity Assay on a TLC Surface

**Figure 3:**
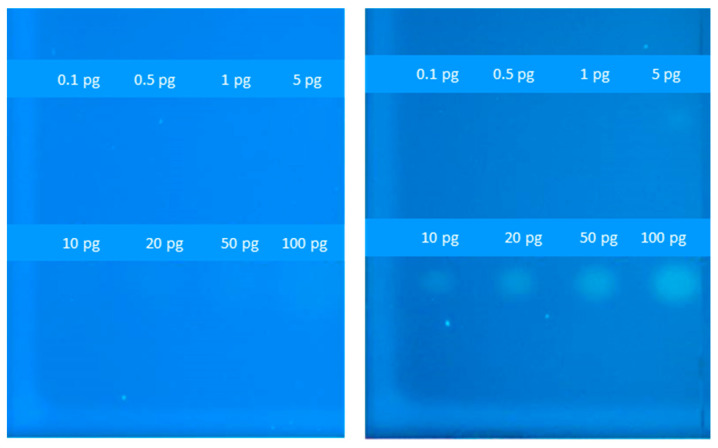
Detection of E2 on HPTLC plates by EGFP-based bioreporters. Different concentrations of estradiol (0.1–100 pg/spot) were applied manually on two HPTLC plates, without subsequent chromatographic development, followed by spraying of sensor strains NAR1 (**left panel**) and NAR5 (**right panel**). Fluorescent EGFP images (excitation 365 nm, emission 565 nm) were acquired by a Fusion FX imaging system (Vilber Lourmat) 18 h post-exposure.

As shown above, strain NAR5 stood out among the five tested bioreporters by displaying both the lowest LOD values and highest fluorescence intensities in the 96-well microtiter plate assay. To further test the functionality of this strain, different concentrations of the model compound E2 were applied on duplicate HPTLC plates, which were then coated by a sprayed-on thin layer of either the NAR1 or the NAR5 strains. An image of the two plates, displayed in Figure 3, clearly indicates the enhanced sensitivity of NAR5, which visibly responded to a spot of 10 pg E2. In the NAR1 control, even 100 pg failed to induce a visible signal.

### 3.3. Detection of Estrogenic Activities in Wastewaters

**Figure 4:**
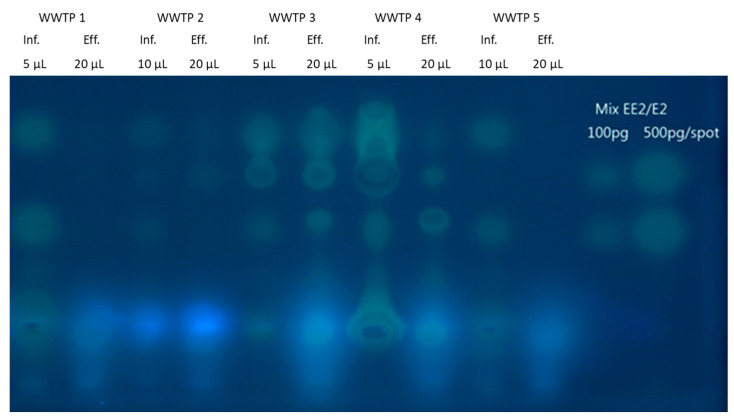
Detection of estrogenic activity in influent and effluent extracts from five wastewater treatment plants. Influent and effluent samples were enriched by solid-phase extraction (SPE) at 200- and 500-fold, respectively. Extracts were applied on the HPTLC plate in volumes of 5 µL or 10 µL for influent samples and 20 µL for effluents. An estrogen mix consisting of EE2 and E2 (100 and 200 pg/spot) was separated on the same plate for calibration and control purposes. Chromatographic separation was conducted as described in Materials and Methods. The fluorescent EGFP signals were detected using the Fusion FX imaging system (Vilber Lourmat) at excitation and emission wavelengths of 365 nm 565 nm, respectively.

Influent and effluent samples, collected from five municipal WWTPs, were chromatographically separated on an HPTC plate. The estrogenic activity of the different sample fractions was then assayed by spraying strain NAR5 onto the plate surface, and imaging the fluorescence above the different sample components following an 18 h incubation (Figure 4). As a positive control, an E2/EE2 mixture at two concentrations was applied and separated at the two right-hand lanes. Estrogenic activity was observable all samples, influents and effluents alike (Figure 4), indicating that, in most cases, the biological treatment process failed to completely eliminate all traces of contaminants with estrogenic-like activities. A tentative assignment of the individual signals to candidate compounds is possible by comparing the migration distances of the detected spots and the reference compounds. In Figure 4, the upper and lower signal in the reference lanes is caused by EE2 and by the natural E2, respectively. In all influent samples, signals with a similar retention to E2 are visible but are absent from the effluent samples. This is in line with the removal of E2 by conventional wastewater treatment. However, a number of signals indicating estrogenic compounds are visible after treatment, indicating an incomplete removal of these chemicals. Since a normal-phase chromatography was performed, more polar compounds are retained by the stationary phase. Thus, the presence of fluorescence signals with a higher migration distance indicates estrogenic compounds with lower polarity, such as alkylphenols. An identification of individual compounds was beyond the scope of this work, but information gained by the thin-layer chromatography, such as the polarity of a compound, is valuable for a subsequent in-depth effect-directed analysis.

## 4. Discussion

In a previous study [7], we designed and constructed a panel of yeast-based fluorescent sensor strains that harbored several different plasmids designed to detect the presence of chemical targets with estrogenic and androgenic activities. The use of fluorescent proteins as reporter elements allows for simultaneous detection by spectral imaging for each of the fluorescent proteins. Combined with thin-layer chromatographic separation, such EDC-responsive sensor strains allow for an efficient and focused risk assessment [7]. The advantage of this approach compared to a classic compound-based risk assessment is the integrated assessment of all potentially estrogenic compounds in a sample, rather than only regulated target chemicals. For the monitoring of surface water, the use of effect-based methods is discussed [23,24] in line with a further development of the water framework directive using the output of effect-based methods as a sum parameter directly focused on the presence of unwanted biological effects, such as endocrine disruption. Effect-based methods are already implemented in the German wastewater ordinance [25] to monitor treated wastewater for unwanted effects, such as the inhibition of the growth of algae and fish toxicity. The methodological concept described herein might be integrated in such monitoring approaches. Using an estrogenic-targeted sensor strain as a baseline, we describe the successful enhancement of its performance as manifested in the E2 detection sensitivity.

Four complementary strategies were employed to achieve this aim, three of which targeted the plasmid structure, including the elimination of the superfluous expression of the previously employed *luxA* reporter gene, adding an additional EGP gene, and changing the promoter driving EGFP expression. The fourth approach involved introducing three efflux mutations into the host strain, which was expected to limit the removal of harmful molecules and thus increase their intracellular concentrations.

One of the targeted sites for sensor improvement was the ADH1 promoter, the driver of EGFP expression in the original pNAR1 plasmid. ADH1 is a strong constitutive promoter, the inactivity of which in the absence of the target ligand was potentially ensured by an upstream hairpin structure formed by palindromic sequences of hormone response elements [13]. However, we have noted that the hairpin structure was not always sufficiently tight to fully inactivate the strong promoter. We have therefore substituted the ADH1 promoter with a constitutive weaker and shorter CYCmin promoter [6]. In this construct (NAR4), both detection sensitivity (as apparent from the LOD value) and signal intensity were improved compared to the WT strain (NAR1), as was evident in both a 96-well plate assay (Figure 2 and Table 2) and in the HPTLC assays (Figure 3). Further improvement was achieved by modifying the host strain rather than the reporter plasmid. Deletion of three membrane transporters related to drug uptake in strain NAR5 has apparently led to intracellular accumulation of the target materials [26], leading to the induction of the reporter genes at low external concentrations. Indeed, as summarized in Table 2, this sensor strain exhibited the highest signal intensity and response ratio, as well as the lowest detection limit of 8 +/− 1 ng/L.

Fluorescent yeast recombinant bioreporters for the detection of endocrine disruptors with the GFP system were tested by Bovee et al. [27] using the classic 96-well plate method, with a reported E2 detection limit of 27 ng/L, similar to our “WT” NAR1 strain. Detection sensitivity similar to the one reported herein was reported by Sievernich et al. [28], who also constructed fluorescent yeast-based bioreporters harboring two plasmids, one containing the human estrogen receptor (hER) and the second containing the yEGFP gene under the control of a CYC1 promoter with three EREs. The E2 LOD of this strain in a 96-well assay was 5 ng/L.

Chamas et al. [29] applied an HPTLC assay using genetically modified *Arxula adeninivorans* yeast strains containing either a human estrogen, androgen or progesterone receptor and a different fluorescent protein for each target group. The 10 pg E2 detection threshold reported here for strain NAR5 is similar to but higher than the 7.5 pg reported in that publication. A planar bioassay employing a bioluminescent estrogenic sensing variant of the same yeast species [30] yielded an E2 EC_50_ value of 1 ng, 100-fold higher than the LOD reported here for the same compound. It should be pointed out, however, that the yeast chassis employed in the current study is *Saccharomyces cerevisiae*, which also serves as the basis for the yeast estrogen screen (YES) assay [31], now recognized by regulatory agencies worldwide [32]. The generic approach presented here for detection sensitivity enhancement, generated from a molecular biology perspective, may potentially be employed for the construction of future generations of sensor strains.

The spray-on technology we have employed for applying the yeast bioreporters on the HPTLC surface allows for the control of the thickness of the suspension layer; this promotes the formation of clear and sharp migration bands as opposed to the immersion procedure that may smear the bands on the HPTLC plate. Recently, Beatz et al. [14] performed a comparison between two yeast cell application methods, immersion and spraying. In this work, they reported that the sensitivity in both methods was similar. The advantage of the spraying method is the reduced amount of yeast cell needed for the EDC activity.

The combination of chemical separation by HPTLC and an effect-based assay by the yeast biosensors allows for the detection of broad concentrations of model hormones on a HPTLC plate, as well as the separation of environmental samples and the detection of elements exhibiting hormonal activity within these samples. The robustness of the strains in identifying potential estrogen activity in samples was demonstrated by the characterization of influent and effluent samples from wastewater treatment plants, concurrently with model compounds applied on the same plate. The use of reporter strain NAR5, combining the benefits of both plasmid- and host-related modifications, provided a potential picture of the presence of estrogen-like compounds within the applied wastewater sample before and after treatment.

The HPTLC application of the effect-based assay approach presented herein may provide at least a partial answer to the increasing demand for more efficient methods for monitoring micro-pollutants in the aquatic environment, and may be used for multiplex planar bioassays addressing a number of biological effects in parallel [33]. To further broaden the applicability and relevance of this method, it would be desirable to extract the resulting bioactive bands and examine their composition by analytical methods. Further improvement of the separation efficiency of environmental samples is required, followed by chemical analysis (LC/MS and GC/MS) of individual bands to identify specific active sample components. Moreover, the construction of yeast cells containing different receptors for the detection of more classes of hormones would be helpful in targeting and monitoring a wide range of potentially harmful contaminants.

## Figures and Tables

**Table 1:** *Saccharomyces cerevisiae* reporter strains used in this study.

Strain	Host	Plasmid	Comment
NAR1	WT	pNAR1	Baseline plasmid [7]
NAR2	WT	pNAR2	*luxA* deletion
NAR3	WT	pNAR3	*luxA* deletion, EGFPx2
NAR4	WT	pNAR4	*luxA* deletion, CYCmin promotor
NAR5	ΔPDR5, ΔSNQ2, ΔYOR1	pNAR4	*luxA* deletion, CYCmin promotor, 3 transporter mutations

**Table 2:** Estrogenicity detection performance summary of all sensor strains.

Sensor Strain:	NAR1	NAR2	NAR3	NAR4	NAR5
Maximal fluorescence * **	4600 ± 17.15	6050 ± 610	13,100 ± 1052	18,550 ± 4830	43,570 ± 10,230
Maximal response ratio *	2.4 ± 0.2	3 ± 0.3	2 ± 0.4	8.4 ± 1.8	22.9 ± 3
LOD (ng/L)	29 ± 3	34 ± 7	38 ± 4	12 ± 1.5	8 ± 1

* Over the course of an 18 h exposure to E2 (200 ng/L); ** Arbitrary fluorescence units.

## Data Availability

The original contributions presented in the study are included in the article and in the supplementary materials; further inquiries can be directed to the corresponding author (sb@mail.huji.ac.il).

## Supplementary Materials

The following supporting information can be downloaded at: https://www.mdpi.com/article/10.3390/bios14040193/s1, Table S1: Oligonucleotide primer sequences used in this study.
