# Supplementary material for: Enhanced Detection of Estrogen-like Compounds by Genetically Engineered Yeast Sensor Strains
Source: Biosensors (Basel). 2024 Apr 15;14(4):193. doi: 10.3390/bios14040193 (PMC11048378; doi:10.3390/bios14040193)
Supplement: Supplementary file 1 [file biosensors-14-00193-s001.zip › biosensors-2906937-supplementary.pdf]

Table S1. Oligonucleotide primer sequences used in this study.

	Primer name	Primer sequence 5' to 3'	Notes
1	PDR5_ kan _FOR	TTTCGTATCCGCTCGTTCGAAAGACTTTAGACAAAAcagctgaagcttcgtacgctgca	PDR5 knockout
2	PDR5_ kan_REV	AtgcggccgcggatctgccggtctcTAGAATTTTGAATTTGGTTAAGAAAAGAAACTTACCAAGATGGAC	PDR5 knockout
3	SNQ2_kan _FOR	ATAGAATAACACAGCTACCAAAATACGTAAAGAGAATTCAagctgaagcttcgtacgctgcagg	SNQ2 knockout
4	SNQ2_kan_REV	AAAGGCAGATGAATGCACAAAATGTTAAGTTATCTGAAGCCCACAccgcggccgcataggccact	SNQ2 knockout
5	Yor1_kan _FOR	TTTATATTCAAAAAGAGTAAAGCCGTTGCTATATACGAATagctgaagcttcgtacgctgcagg	Yor1 knockout
6	Yor1_kan _REV	TTTATATTATTTGTTGCATGATTTTTCTCTTTTATTTccgcggccgcataggccact	Yor1 knockout
11	ADH1_ SpeI _EGFP_FOR	ACTCCCCGGGACTAGTTTAATTAACATGTCAAAA	SpeI
12	ADH1_Kpn1_EGFP_REV	GCTCGGTACCAATCTAGGCGCGCCTTACTTGTATAATTCA	KpnI
13	GPD_SalI_EGFP_FOR	TTTTTTGTCGACCCTTAGTTACTTGTATAATTCATCCA	SalI
14	GPD_NotI_EGFP_REV	TTTTTGCGGCCGCGGACTCTCTATGTCAAAAGGCGAGGAACT	NotI
19	CYC1_EGFP_2_rev	TGTTAATTAAttctttACTAGTCCCGGGtattaatttagtgtgtgtatttgtgtttgcgtgtctataga	NEBuilder assembly
20	CYC1_EGFP_2ERES_FOR	CTTGCATGCCTGCAGGTCGACCAGGTCACTGTGACCTCTCGAGGTCACTGTGACCTG<br>CCGGCtcgagcagatccgccaggcg	NEBuilder assembly
